# Mahatma Gandhi–Doerenkamp Center for Alternatives to Use of Animals in Life Science Education

**DOI:** 10.4103/0976-500X.72353

**Published:** 2010

**Authors:** Mohammad Abdulkader Akbarsha, Shiranee Pereira

**Affiliations:** *Bharathidasan University, Tiruchirappalli 620 024, India*; 1 *People for Animals, Chennai, India*

Very few men have done so good to fellow creatures as Mahatma Gandhi. He said, ‘We should not inflict cruelty on even the meanest of creatures. I also will have to answer for this in the court of the Almighty.’ No single person ever in the history contributed so much wealth to protection of animals as Ms. Hildegard Doerenkamp from Germany, the founder of Doerenkamp-Zbinden Foundation. It is great that a centre in the names of these two towering personalities, to propagate the message of ‘no animal kill’ in education, research and testing, has come up at Bharathidasan University, Tiruchirappalli, India. This University named after the great revolutionary Poet, Bharathidasan, who vowed ‘To create a brave new world,’ has come to be blessed with locating this Center here. It was a red letter day in the history of this University and Doerenkamp-Zbinden Foundation, Switzerland, when these two parties entered into a Memorandum of Understanding to establish the ‘Mahatma Gandhi–Doerenkamp Center for Alternatives to Use of Animals in Life Science Education,’ on July 13, 2009, the Center was launched on July 15, 2009 and formally inaugurated on the 141st birth anniversary of Mahatma Gandhi, October 02, 2009.

If William Russell and Rex Burch were the ones to sensationalize the world with their book *The Principles of Humane Experimental Technique*, published in 1959, where in they craved for a humane approach to animals in experimentation and thus introduced the concept of 3Rs(Refinement, Reduction and Replacement), D. Smith was the first to introduce the term ALTERNATIVES, in 1978, to convey the concept of 3Rs; it was Jennifer Graham, a brave 15-year-old girl from California, who in 1987 refused to dissect an animal and sued her school district seeking an alternative study option, whereupon California, USA, granted that right to all high school students, to be followed up by other states in the US and later other countries as well and revolutionized the history of animal dissection and vivisection. In as much as humane approach to animals in education, research and testing has been taken up seriously in the developed countries, the developing countries are yet to catch up. The situation in India, the country with the second largest population in the world, has been dismal.

There was an awakening by the turn of the century when three organizations, People *for* Animals (India), I-CARE (India) and InterNICHE (UK) began a campaign for non-animal methods of teaching and learning of life science, biomedical science and veterinary science. The effort came to be rewarding because these organizations roped in the service of the academicians, Prof. M.A. Akbarsha from Bharathidasan University; Dr. M.C. Sathyanarayana from AVC College, Mannampandal, Mayiladuthurai, a college affiliated to Bharathidasan University; Dr. Ramakrishna, a Professor of Veterinary Science from Chennai; Dr. R. Raveendran, a pharmacologist from JIPMER, Pondicherry; Dr. Syed Ziaur Rahman, another pharmacologist from Aligarh Muslim University; and others, in their endeavor. More importantly, they targeted the teachers, the ones who are the key role players in the academic curricular decisions, to bring in a change. A workshop was conducted on the September 25, 2001 at AVC College, Mannampandal, Mayiladuthurai, Tamil Nadu, organized by Dr. M.C. Sathyanarayana, where Prof. M.A Akbarsha delivered the key note address. The participants, all college teachers, were introduced to the non-animal methods of learning anatomy in Zoology. From then on, great many programs were conducted in many places in the country, but were all done in piece-meal, without a clear direction. A national congress was also conducted in Chennai by I-CARE in 2006. One admirable achievement of this drive was sensitization of the University Grants Commission, New Delhi, the Regulatory Authority of Higher Education in India, by People *for* Animals, supported by I-CARE, Chennai and Prof. M.A. Akbarsha, which resulted in the origin of an epoch-making letter to all Universities in India requiring curtailment of use of animals in Zoology teaching and learning. But little was done thereafter since the efforts were from certain unorganized sectors and freelancers.

Prof. M.A. Akbarsha–Dr. M.C. Sathyanarayana combine approached the change in a different approach as well. Being senior teachers and scientists of Zoology, they were members of the curriculum boards in several Universities. Taking advantage of this position, they worked in the Curriculum Boards and made effort to change the Zoology curriculum, such that animal dissection as an aspect of animal anatomy laboratory exercise was greatly reduced and even dropped in some universities/programs. The most exciting outcome was from Bharathidasan University, where all major dissections were dropped from the curriculum for undergraduate as well as postgraduate programs and in the place learning of animal anatomy using CD-Roms was introduced for the first time in the country. This came to be acclaimed as the ‘Bharathidasan University Model’ (Akbarsha, 2007). In the mean time, *PETA* India worked with the Pharmacy Council of India and caused a decision that where alternative methods for pharmacological testing are available, the *in vivo* testing protocols need not be practiced. Later came the revision of the guidelines of Indian Medical Council, wherein alternatives in the place of live experiments are encouraged. Thus, the roles of I-CARE, People *for* Animals, *PETA* India and InterNICHE are commendable. Yet, the vastness of the country, the variety in the higher education and the heterogeneity of the religious, linguistic and cultural heritages of the people made the task enormous for such unorganized workers.

At this time, there was an initiative from the Chennai chapter of People *for* Animals to bring in aggression in the campaigning of non-animal methods in Life Science and Biomedical Science education in India. Because Bharathidasan University had already established a mark in the campaign, spearheaded by Prof. M.A. Akbarsha and Dr. M.C. Sathyanarayana, it was proposed that a National Center for Alternatives be established at this University, with Prof. M.A. Akbarsha in the helm of affairs. Before Funding Agencies in India could be approached, coming to know about the initiative, Doerenkamp-Zbinden Foundation offered to participate in the venture, deciding for the first time to expand its activity to outside Europe and USA. It was proposed that a National Center for India be established, in a tripartite collaboration of DZ Foundation, Bharathidasan University and People *for* Animals, Chennai, wherein the Foundation will bear the entire cost, including a chair and a building proposed in a budget; P*f*A will render moral and counseling support and Bharathidasan University will house and run the Center. Thus came up the MoU, signed on July 13, 2009, providing for establishment of the Center.

The Center is named after Mahatma Gandhi and Ms. Hildegard Doerenkamp. Mahatma Gandhi was the great leader of India during the most gruesome period of Indian history. Central to Mahatma Gandhi’s vision was an impassioned conviction that at the heart of all life, there is ‘Truth’ which sustains all creation; a ‘Truth’ which demands a personal response from each individual. He saw ‘Truth’ as a truth present in every person. In particular, he held nonviolence as a basic tenet of this ‘truth,’ a positive force that can bring about fundamental change at all levels. For Gandhiji ‘nonviolence’ was the discovery of a new kind of power. It is a well-known fact that Gandhiji not only played a major role in India achieving its independence, but taught a philosophy which has universal applicability. The core of that philosophy is the search for truth through nonviolence – ‘Ahimsa.’ Gandhiji taught respect for animals as well as humans, a non-exploitative relationship to the environment, the elimination of poverty, the limitation of personal wealth and possessions and nonviolence applied at all levels of relationships, be it man to man, man to animal, or man to environment. According to him, ‘The greatness of a nation and its moral progress can be judged by the way its animals are treated. He said ‘I hold that the more helpless a creature, the more entitled it is to protection by man from the cruelty of man,’ and ‘I abhor vivisection with my whole soul. All the scientific discoveries stained with innocent blood I count as of no consequence.’ Ms. Hildegard Doerenkamp dedicated her entire wealth toward the cause of animals and was responsible for establishment of the DZ Foundation, along with Dr. Gerhard Zbinden. This Foundation, to begin with, has been an extending support for discovery of alternatives and conferring awards for outstanding work on alternatives. Later, it started funding to establish Centers and Chairs on alternatives. It has Chairs at Johns Hopkins University, USA; University of Geneva, Switzerland; University of Konstanz, Germany; and Utrecht University, The Netherlands. The Foundation is also the nucleus which publishes the journal on alternatives, *ALTEX*.

Prof. M.A. Akbarsha, basically a teacher of Zoology and Animal Science for more than three decades, also a scientist who uses *in vitro* tools, has been declared the Director of the Center and also the Gandhi-Gruber-Doerenkamp Chair for Alternatives in Life Science and *in vitro* Toxicology. He is adequately supported in this campaign by Dr. Shiranee Pereira (I-CARE, Chennai), Dr. M.C. Sathyanarayana (Chennai), Dr. Krishan Kant Sharma (Ajmer), Dr. R. Raveendran (Pondicherry), Dr. P. Natarajan (Ambo University, Ambo, Ethiopia) and others.

Following are the mandates of the Center:

- The Center will endeavor to advance the concept of ‘humane science’ and implement the concept of the 3Rs in education, research and testing, in accordance with Indian legislations, the international ‘Declaration of Bologna,’ and international advances in the science of alternatives.
- The Center will work to create a strong and positive presence for alternatives to the use of animals in experimentation/testing in India and proactively work to blend life science education with the Gandhian philosophy of nonviolence.
- The Center will evolve a national program for humane education in teaching and research.

The centre will work to:

- Develop a national humane education program for all universities/colleges/national research institutes as part of their life science curriculum/research, which will seek to blend the Gandhian philosophy of nonviolence with life science education/research.
- Develop strategy for the implementation of the 3Rs in academic and research/testing laboratories.
- Conduct courses on Humane Science and Alternatives in the Use of Animals in Education and Research in affiliation with universities of repute like the Oxford University Centre of Animal Ethics, UK and CAAT, Johns Hopkins University, USA, etc., for both Indian and international students.
- Support by way of funds and expertise high-quality research that advances the 3Rs for development of pedagogic tools computer modeling for teaching, drug development, basic biomedical research, product testing, etc.
- Provide expertise and guidance on the 3Rs and laboratory animal welfare to the teaching/scientific community by developing a range of resources, including guidelines and training material (e.g., CDs), organizing working groups, workshops, symposia, etc.
- Liaise with national educational councils like MCI, VCI, PCI, UGC, AICTE, etc. and state education departments for curricular developments to promote the use and knowledge of alternatives.
- Liaise with regulatory bodies for the acceptance of alternative methods in product testing.
- Establish a state of the art tissue culture laboratory and library of alternatives, which will help train scientists in the use of alternatives. The former could also help generate revenue for the Center by way of product testing on payment basis.
- Help create virtual learning Centers in universities.
- Organize national level training workshops, seminars, national congresses, etc., to propagate the *in vitro/in silico* methods and other alternatives, with international expertise/collaboration and to train Institutional Ethics Committee members.

Following are the mission goals:

- To establish a library of alternatives.
- To provide for lending of alternatives.
- To offer training in use of alternatives.
- To motivate the teachers not only to get prepared for introduction of alternatives in teaching and research, but to work in the Curriculum Boards to change the course content so as to replace animals in dissection, experiments and testing with appropriate alternative modalities.
- To conduct research to develop newer alternatives.
- To work for application of alternatives in life science and biomedical science research.
- To conduct teaching programs in ‘Ahimsa and Alternatives’ and ‘*In Vitro* Toxicology’

The following facilities are being built:

- A repository of dissection CDs, videos, mannequins, models, etc.
- A state of the art computational lab.
- *in vitro* testing facility and a cell line bank.
- Bioinformatics tools such as predictive toxicology (Q)SAR, Read Across, ebTrack, etc., for *in silico* analysis.

How does the Center intend to go about reaching the goals:

- To conduct workshops at identified places to train the University and College teachers to use the dissection alternatives.
- To lend dissection alternatives to the institutions further, to encourage changing to the newer pedagogy of teaching animal anatomy.
- To train the teachers and researchers in *in vitro* and *in silico* alternatives.
- To organize national and international seminars and conferences to popularize the concept of alternatives.

**Table:** The task is enormous. As already said, India is a vast country with a huge population.

Area	:	3.3 million sq km
Number of States	:	28
Number of Union Territories	:	7
Population	:	1186.2 million
Ethnic groups	:	Indo-Aryan 72%, Dravidian 25% Mongoloid and others 3%
Number of languages spoken	:	21 (scheduled languages)
Number of Central Universities	:	39
Number of State Universities	:	131
Number of Deemed Universities	:	127
Number of Colleges	:	6289
Number of Science Colleges	:	1868
Number of Medical Colleges	:	274

Yet, the Center is optimistic. The goal of non-animal methods in teaching, research and testing as a national policy and practice is expected to be reached soon.

